# From Confinement to Autonomy: Improvement of Extreme Aggressiveness in ASD After Deep Brain Stimulation

**DOI:** 10.1192/j.eurpsy.2026.11759

**Published:** 2026-07-31

**Authors:** P. Barcelo, C. Hernandez, C. Velasquez, K. Oyola, M. Pinedo, L. Gutierrez, S. Perneth

**Affiliations:** 1 Atlántico, Universidad Simón Bolívar; 2 Atlántico, ESE Universitaria del Atlántico, Barranquilla, Colombia

## Abstract

**Introduction:**

Severe aggressiveness in autism spectrum disorder (ASD) with intellectual disability represents a complex therapeutic challenge. In refractory cases, deep brain stimulation (DBS) emerges as a functional psychosurgical alternative capable of modulating circuits involved in impulsivity and aggressive behavior, offering an opportunity to improve quality of life.

**Objectives:**

To describe the clinical evolution, impact on quality of life, and recovery of dignity in a patient with ASD and extreme aggressiveness, before and after the implementation of deep brain stimulation (DBS).

**Methods:**

A 17-year-old male with ASD level 3, intellectual disability, and severe behavioral disorder characterized by auto- and hetero-aggressiveness, and global insomnia. Since the age of 4, he had received multiple psychopharmacological treatments without sustained response, remaining under preventive restraint due to the risk of aggression toward caregivers. He experienced multiple psychiatric hospitalizations; during his prolonged stay, violent and agitated episodes persisted despite pharmacological adjustments. DBS was performed using a stereotactic system with quadripolar electrodes and an implantable pulse generator targeting the posteromedial hypothalamic region. The KidsLife Scale was applied before and one year after the intervention to assess changes in functionality and quality of life.

**Results:**

As shown in Figure 1, KidsLife results demonstrated domain-specific variations. The patient exhibited a significant reduction in aggressive crises, greater emotional stability, and a marked decrease in the need for physical restraint, achieving participation in supervised activities and showing enhanced affective interaction with his environment.

**Image 1:**
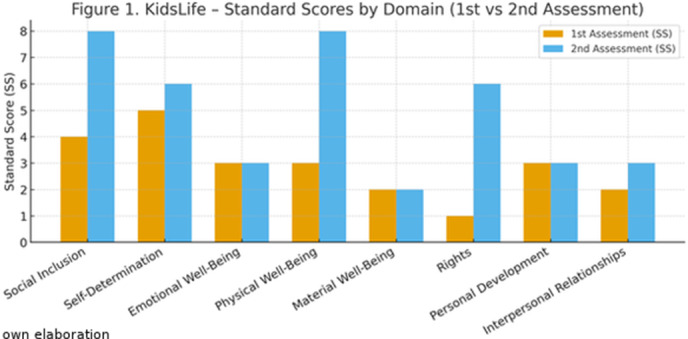
Image 1: Long description.

**Conclusions:**

This case illustrates how deep brain stimulation can improve the course of refractory aggressiveness in ASD, enabling the transition from containment to connection, with improved physical well-being and reduced caregiver burden. The functional and emotional improvements observed highlight the value of integrated neurofunctional approaches aimed at restoring dignity and quality of life for both the patient and the family.

**Disclosure of Interest:**

None Declared

## Long descriptions

### Image 1: Long description

A grouped bar chart titled KidsLife - Standard Scores by Domain. The y-axis is labeled Standard Score (S S) with a scale from 0 to 8. The x-axis lists eight domains. A legend in the top right identifies orange bars as 1st Assessment (S S) and blue bars as 2nd Assessment (S S).

From left to right, the data pairs are:

* Social Inclusion: 1st assessment is 4, 2nd assessment increases to 8.

* Self-Determination: 1st assessment is 5, 2nd assessment increases to 6.

* Emotional Well-Being: Both assessments are equal at 3.

* Physical Well-Being: 1st assessment is 3, 2nd assessment increases to 8.

* Material Well-Being: Both assessments are equal at 2.

* Rights: 1st assessment is 1, 2nd assessment increases to 6.

* Personal Development: Both assessments are equal at 3.

* Interpersonal Relationships: 1st assessment is 2, 2nd assessment increases to 3.

Navigate back to Image 1:.

